# Targeting metabolism of breast cancer and its implications in T cell immunotherapy

**DOI:** 10.3389/fimmu.2024.1381970

**Published:** 2024-04-12

**Authors:** Jialuo Zou, Cunjun Mai, Zhiqin Lin, Jian Zhou, Guie Lai

**Affiliations:** ^1^ Department of Breast Disease Comprehensive Center, First Affiliated Hospital of Gannan Medical University, Gannan Medical University, Ganzhou, Jiangxi, China; ^2^ Department of Immunology, International Cancer Center, Shenzhen University Health Science Center, Shenzhen University, Shenzhen, Guangdong, China

**Keywords:** breast cancer, T cell immunotherapy, metabolic reprogramming, immune escape, glucose, amino acid, lipid

## Abstract

Breast cancer is a prominent health issue amongst women around the world. Immunotherapies including tumor targeted antibodies, adoptive T cell therapy, vaccines, and immune checkpoint blockers have rejuvenated the clinical management of breast cancer, but the prognosis of patients remains dismal. Metabolic reprogramming and immune escape are two important mechanisms supporting the progression of breast cancer. The deprivation uptake of nutrients (such as glucose, amino acid, and lipid) by breast cancer cells has a significant impact on tumor growth and microenvironment remodeling. In recent years, in-depth researches on the mechanism of metabolic reprogramming and immune escape have been extensively conducted, and targeting metabolic reprogramming has been proposed as a new therapeutic strategy for breast cancer. This article reviews the abnormal metabolism of breast cancer cells and its impact on the anti-tumor activity of T cells, and further explores the possibility of targeting metabolism as a therapeutic strategy for breast cancer.

## Introduction

Breast cancer, the frequently occurring cancer in women, remains a public-health issue on a global scale. Statistics estimate about 1 million new cases of breast cancer diagnoses and 370,000 deaths annually around the world. In China, the incidence rate of breast cancer reaches 21.6 per 100 000 (1, 2). Particularly, breast cancer patients are trending younger in China, with an average age of 45-55 at diagnosis (3). Currently, the therapeutic options available for breast cancer include surgery, chemotherapy, radiotherapy, endocrine therapy, and targeted therapy. However, these existing therapies are insufficient to realize desirable efficacy due to side effects and drug resistance (4, 5). For the effective management of breast cancer, efforts should be made not only to reduce the morbidity and disease-related mortality, but also to control the incidence of complications related to treatment. Therefore, innovative therapeutic approaches for breast cancer are urgently needed.

Genetic variations or mutations within cells are increasingly identified as important predisposing factors to a variety of cancers. The aberrant expression of oncogenes and tumor suppressor genes profoundly alters cellular metabolism, providing the driving force for cancer pathogenesis. Metabolic reprogramming is now recognized as a hallmark of cancers and supports cancer cells to survive and proliferate rapidly in a malnourished tumor microenvironment (6–8). The overlapping metabolic reprogramming in cancer cells is a putative determinant of cancer initiation and progression (9). At present, cancer cell metabolic abnormality is considered a promising research field.

In recent years, immunotherapy has been proposed as the fourth cancer therapy after surgery, radiotherapy, and chemotherapy (10). A growing body of evidence suggests that immunotherapy is a powerful clinical therapeutic strategy for breast cancer (11), melanoma (12), lung cancer (13), lymphoma (14), and prostate cancer (15), largely improving the quality of life and prognosis of patients (16, 17). Despite revolutionizing immunotherapy, the overall response rate remains low and merely a small proportion of patients can benefit from it (18). Poor efficacy and advanced drug resistance remain obstacles to immunotherapy for breast cancer. Against this backdrop, the metabolic characteristics of cancer cells may present promising targets for improving the efficacy of immunotherapy.

## Subtypes of breast cancer

**Figure 1:**
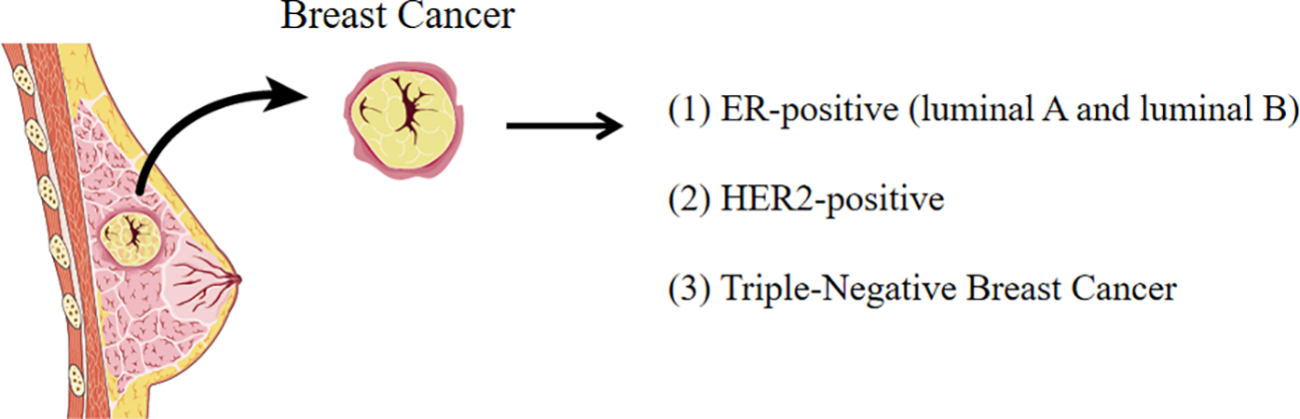
The subtypes of breast cancer. Breast cancer is characterized as four molecular subtypes based on the molecular markers (ER, PR, and HER2).

Breast cancer can be characterized as four molecular subtypes based on the presence or absence of molecular markers such as estrogen receptor (ER) or progesterone receptor (PR) and epidermal growth factor receptor 2 (ErbB2, also known as HER2): namely, luminal A, luminal B, HER2+, and triple-negative breast cancer (TNBC) (19). ER-positive breast cancer, including luminal A and luminal B, is the most common subtype of breast cancer, accounting for 70% breast cancer cases (20). HER2-enriched subtype accounts for nearly 15% of breast cancers (21). HER2 is a crucial oncogenic driver that promotes breast cancer cell proliferation but prevents cell apoptosis. TNBC, as the name implies, tests negative for estrogen receptor, progesterone receptor, and HER2, which is usually (but not always) a basal breast cancer with the worst prognosis (22). Each breast cancer subtype has different proliferation rates, invasion and metastasis abilities, metabolic phenotypes, and genotypes as shown in **Figure 1**.

Through the combination of transcriptomics and metabolomics analysis, researchers have identified changes in metabolite levels and related metabolic pathways in different subtypes. This comprehensive research method enables us to gain a deeper understanding of the specific metabolic changes in different breast cancer subtypes, which contributes to revealing the pathogenesis mechanism of cancers more accurately and providing important evidence for developing personalized treatment schemes targeting specific metabolic pathways. These findings not only help us understand the complexity of cancer, but also provide new directions and ideas for future cancer treatment research (23).

## Metabolic reprograming against immunotherapy

**Figure 2:**
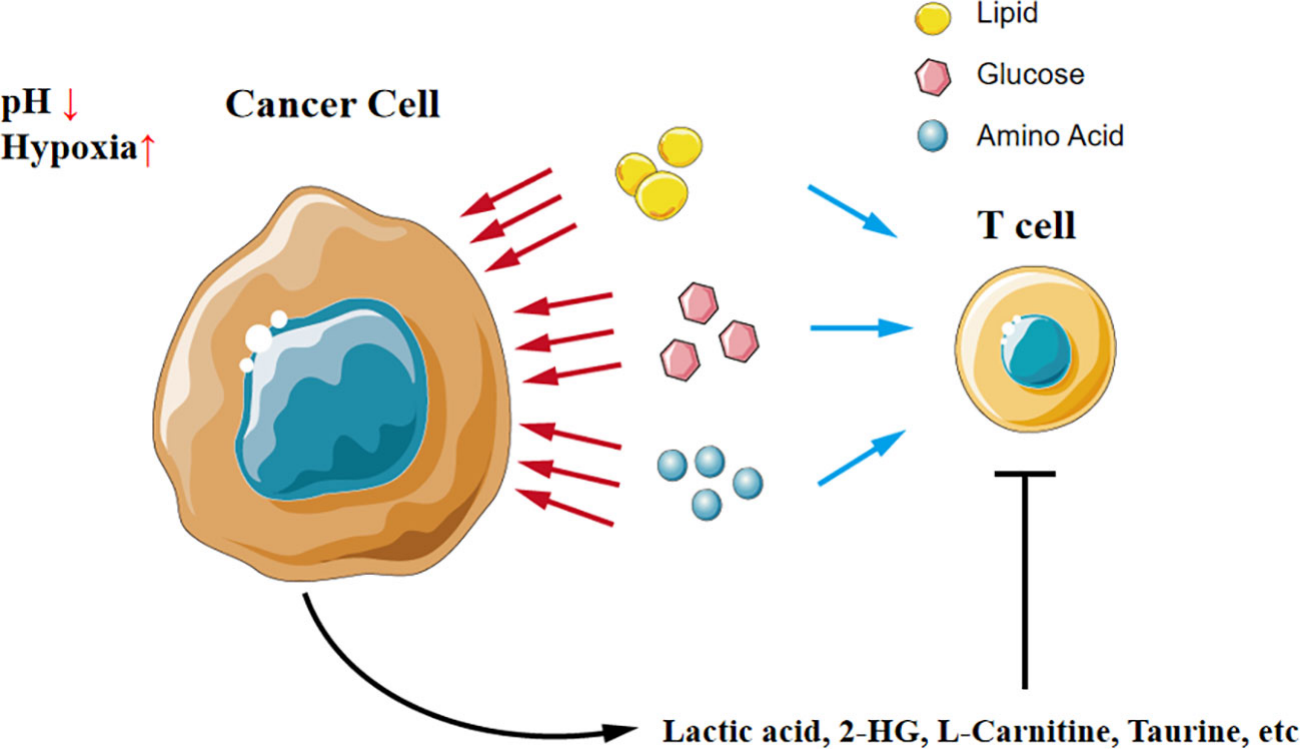
A tug-of-war between cancer cells and T cells in the microenvironment. Cancer cells competitively uptake nutrients like glucose, amino acid, and lipid, which results in T cells in the microenvironment lacking nutrients to support function. Additionally, cancer cells secrete oncometabolites and directly compromise T cells.

Tumor cells remodel the tumor microenvironment by reprograming the metabolism in two manners: (1) competitive uptake of essential nutrients and (2) production of oncometabolites as shown in **Figure 2**.

T cells compete fiercely with tumor cells for nutrients in the tumor environment, just like a tug-of-war between tumor cells and T cells. Transporters of key nutrients like glucose and amino acids are highly expressed in tumors, leading to a shortage of these substances in the local environment. For instance, tumor cells outperform T cells for competing methionine, thus disrupting methionine metabolism in CD8^+^ T cells and impairing T cell immunity (24).

Simply defined, oncometabolites are conventional metabolites that have pro-oncogenic functions when aberrantly accumulated, thereby driving tumorigenesis, development, and metastasis. The accumulation of oncometabolites, such as D-2HG, is often attributed to the mutations in genes encoding enzymes of the citric acid cycle, and these genetic mutations are commonly considered driving factors for tumor development (25, 26).

## Glucose metabolism

**Figure 3:**
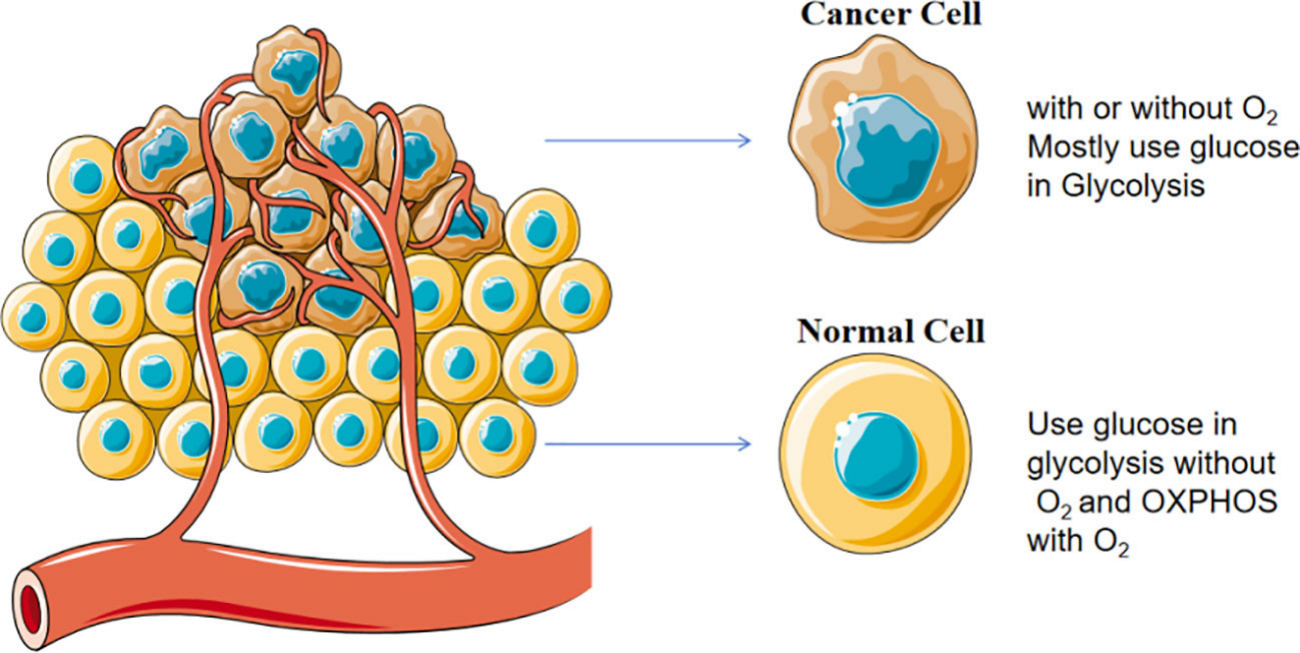
Warburg effect in cancer cells. Cancer cells prefer to consume glucose through glycolysis in the presence of sufficient oxygen and fully functional mitochondria.

Mitochondria are the energy-producing organelles and generate adenosine triphosphate (ATP) for supporting the key functions of cells by 2 metabolic pathways, namely glycolysis and oxidative phosphorylation (OXPHOS). Even in the presence of sufficient oxygen and fully functional mitochondria, tumor cells still prefer to produce substantial amounts of energy through a high glycolytic metabolism, a metabolic pattern known as the “Warburg effect” (27) as shown in **Figure 3**. Although the ATP produced by glycolysis is less than that produced by OXPHOS, this process is faster and more conducive to providing energy for the rapid proliferation of tumor cells (28). In aerobic glycolysis, glucose transporters are responsible for the uptake of glucose into tumor cells and then give rise to the end product pyruvate. *TP53* gene mutation frequently occurs in breast cancer (29), resulting in normal glucose metabolism toward aerobic glycolysis (30).

## Glucose transporters

Two families of glucose transporters have been identified in human: facilitative glucose transporters (GLUT) and Na+-coupled glucose transporters (SGLT). Most cancer cells increase glucose uptake by up-regulating glucose transporters (31). It is reported that GLUT1-6 and 12 are highly expressed in breast cancer (32). The high expression of GLUT1 in breast cancer is associated with advanced histological grade, low differentiation, and consequently poor prognosis (33). Thus, GLUT1 is considered an oncogene in breast cancer (34, 35). Inhibition of GLUT1 by genetic editing or pharmacological inhibitor BAY-876 can restrain the growth of TNBC cells exhibiting high glycolysis rate and low OXPHOS rate (36, 37). GLUT4 plays a key role in glucose uptake of MCF7 and MDA-MB-231 breast cancer cells. GLUT4 shRNA can reduce glucose uptake and induce metabolic reprogramming, redistributing metabolic flux to oxidative phosphorylation. Under hypoxic conditions, loss of GLUT4 can critically repress breast cancer cell proliferation and impair cell viability, verifying the feasibility of inhibiting GLUT4 pharmacologically to induce *in vivo* metabolic reprogramming in cancer models (38).

CD8^+^ T cells are important immune cells that augment anti-tumor immunity. The transition from oxidative phosphorylation to aerobic glycolysis is a hallmark of T cell activation and is considered a metabolic requirement for its proliferation (39, 40). However, high glucose consumption by tumor cells can lead to a decrease in glucose uptake of CD8^+^ T cells, which inhibits CD8^+^ T cell activity and promotes tumor growth. Alterations in glucose uptake of cancer and immune cells via different glucose transporters can predict immunotherapy response (41). Selectively targeting glucose transporters in cancer cells without compromising glucose uptake in immune cells may be a promising therapeutic strategy. In single-cell RNA-seq analysis, cancer cells have the highest expression of GLUT1, while immune cells have the highest expression of GLUT3 (41). Hence, inhibition of GLUT1 may increase glucose levels in the tumor microenvironment and glucose consumption by immune cells, thereby enhancing the efficacy of immunotherapy. Additionally, facilitating the glucose transport capacity in CD8^+^ T cells can also favor immunotherapy. For instance, embedding the CD28 signal domain in the chimeric antigen receptor (CAR) structure promotes T cell glucose uptake, increases glucose transporter GLUT1 expression, enhances CAR-T cell glycolytic activity, and amplifies the effector activity of CAR-T cells (42). This study demonstrates from the perspective of T cells the indispensable role of glycolysis in enhancing the anti-tumor activity of T cells.

Since the competitive uptake of glucose by tumors in the tumor microenvironment inhibits the function of effector T cells, targeting the glycolytic pathway of tumor cells has become a potential therapeutic strategy to improve tumor immunotherapy. 2-Deoxyglucose (2DG) is an analog of glucose that can competitively bind to hexokinase, the rate-limiting enzyme of glycolysis, thereby inhibiting the metabolic pathway of glycolysis. It has been reported that the combination of 2DG and cytotoxic drugs can enhance the activity of immune cells and significantly prolong the lifespan of immunocompetent mice, but does not affect the lifespan of immunocompromised mice (43), which may be related to the restoration of glucose concentration in the tumor microenvironment, but the effect of 2DG itself on T cells has not been emphasized in this study. In addition, it has also been reported that inhibiting glycolysis in CD8^+^ T cells can promote the formation of long-term memory and the anti-tumor function of CD8^+^ T cells (44), but the relevant mechanisms need to be further clarified.

## Glucose metabolism metabolites

Cancer cells including breast cancer cells depend upon aerobic glycolysis to provide the energy they need to survive and proliferate. Lactic acid is an end product of glycolysis. With the contribution of tumor stromal cells, the high level of lactic acid secretion under hypoxic conditions can lead to extracellular acidification (45).

It is now well accepted that increased lactic acid production and the resultant acidification of the tumor microenvironment promote key carcinogenesis processes such as angiogenesis, migration, and metastasis (46). In turn, acidification of the tumor microenvironment obstructs the immune surveillance and response (47). A study has demonstrated that lactic acid is a potent inhibitor of T and NK cell function and survival, leading to tumor immune escape (48). High concentration of lactic acid in the tumor environment can prevent the efflux of lactic acid from T cells, thereby interfering with the metabolism and function of T cells, including chemotaxis and respiratory activity (44). Interestingly, a research team proposes that lactate-lowering mood stabilizer lithium carbonate can inhibit lactic acid-mediated CD8^+^ T cell immunosuppression, indicating that targeting lactic acid metabolism can support cancer immunotherapy (49).

When the pH of the tumor microenvironment decreases to a value specific to tumor masses (pH 6-6.5), human and murine tumor-specific CD8^+^ T lymphocytes lose their immune response to tumor cells, manifested by impaired cytotoxic activity and reduced cytokine secretion, decreased expression of IL-2Rα (CD25) and T cell receptor (TCR), and weakened activation of signal transducer and activator of transcription 5 (STAT5) and extracellular signal-regulated kinase (ERK). If the pH is buffered within the physiological range, T cell function is fully restored (50).

Lactate dehydrogenase (LDH) catalyzes the final reversible step of the glycolytic pathway, reducing pyruvic acid to lactic acid. It is either a homotetramer or a heterotetramer consisting of subunits called “A” and “B”, and their assembly produces five different isoenzymes. Higher LDH expression is associated with poorer prognosis in many types of tumors (51). Of note, the expression of lactate dehydrogenase A (LDHA) in breast cancer tissues is much higher than that in adjacent tissues, and it can maintain cancer stemness and promote the plasticity of breast cancer stem cells (52). Moreover, lactate dehydrogenase B (LDHB) is an essential gene in TNBC. The loss of LDHB inhibits tumor cell proliferation *in vitro* and tumor growth *in vivo*, and breast cancer patients with higher expression of LDHB tend to have poorer clinical outcomes (53). These findings suggest that LDH inhibition may be a promising therapeutic target for breast cancer (54, 55).

High expression of circulating LDH has traditionally been regarded as a marker of poor prognosis in various cancer types, typically attributed to increased tumor burden and cancer metabolism. However, recent evidence suggests that elevated LDH levels may be independent of tumor burden and carry a negative predictive value of immunotherapy (56, 57). Overexpression of serum LDH and presence of liver metastases are dominant predictors of primary cancer resistance to anti-PD(L)1 immunotherapy (58). Scientific evidence published over the past few years has revealed the central role of lactate as an active metabolite. Lactate is no longer solely considered a byproduct of glycolysis, but rather a fuel for the tricarboxylic acid cycle, oxidative phosphorylation, and oncogenic molecules. Lactic acidosis, a hallmark of solid tumor microenvironment, originates from lactate hyperproduction and stimulates tumorigenesis, invasion, and metastasis (59). Lactic acid can promote programmed death-1 (PD-1) expression in regulatory T cells in highly glycolytic tumor microenvironments, such as MYC-amplified tumors. Therefore, the activation of PD-1-expressing Treg cells may lead to the failure of PD-1 blockade therapy (60, 61). Lactic acid alters the tumor microenvironment into a low pH state, which is harmful to T cells. However, the impact of lactate on cancer cells and immune cells can be extremely complex, which is further complicated by the acidic protons, a byproduct of glycolysis. Subcutaneous injection of sodium lactate, rather than glucose, into mice bearing transplanted MC38 tumors inhibits CD8^+^ T cell-dependent tumor growth. Single-cell transcriptomics analysis reveals an increased proportion of CD8^+^ T cells expressing the stem-like transcription factor TCF-1 among CD3^+^ cells within the tumor (62). These findings provide evidence for the intrinsic role of lactate in anti-tumor immunity independent of the pH-dependent effect of lactic acid.

Monocarboxylate transporters (MCTs) catalyze the proton-linked transport of monocarboxylates, such as lactate and pyruvate, across the biological membrane. Highly malignant tumors rely heavily on aerobic glycolysis, so it is necessary to export lactate out of the tumor microenvironment through MCTs to maintain a strong glycolytic flux and prevent the tumor from being “pickled to death”. Most breast cancer cell lines express MCT isoforms (MCT1, MCT2, MCT4) (63), while MCT3 expression is significantly downregulated in breast cancer tissues (64). The expression of MCT1 is closely related to the metabolism and proliferation of breast cancer cells. High expression of MCT1 is usually indicative of malignancy degree and poor prognosis in breast cancer, suggesting the potential of MCT1 as a therapeutic target. Inhibition of MCT1 can interfere the energy metabolism of tumor cells, thereby curbing the growth and metastasis of tumors (65). MCT4 is overexpressed in HER2-positive breast cancer and related to poor prognosis. MCT4 supports pH maintenance, lactate secretion, and glucose metabolism in breast cancer cells. The consumption of MCT4 reduces the growth ability of breast cancer cells in three-dimensional matrix or multilayered spheres (66). Moreover, enrichment analysis reveals that MCT4-related genes are implicated in immune and metabolism-related biological processes, such as the adaptive immune system. MCT4 may play a crucial role in maintaining the tumor immune microenvironment (TIME) through metabolic reprogramming. Consequently, enzymes involved in the glycolytic pathway (MCT4, PKM2, and HK3) emerge as potential new targets for modulating the TIME and enhancing the efficacy of immunotherapy (67–69).

## Amino acid metabolism

Amino acids are essential nutrients for cancer cells. As cancer cells cannot fully synthesize essential amino acids and some non-essential amino acids to support rapid proliferation, they must acquire amino acids from the surrounding environment. To obtain the amino acids required for proliferation, high levels of amino acid transporters are expressed on the surface of cancer cells. Interestingly, breast cancer cells limit the use of amino acids for cell proliferation according to the availability of amino acids, which depends on the estrogen receptor status (70).

Amino acids are also indispensable nutrients for immune cells. The activation, differentiation, and function of T cells largely depend on the transport and metabolism of amino acids. Naive or resting T cells express relatively low levels of amino acid transporters, and selective amino acid transporters are upregulated within a few hours of T cell activation (71). However, tumor cells compete with T cells for extracellular amino acids by increasing the expression of amino acid transporters, resulting in amino acid shortage, which in turn impairs the proliferation, survival, and effector function of T cells. In addition, tumor cells also release some amino acid-related downstream metabolites with immune regulatory properties in the tumor microenvironment, which can directly disrupt the function of T cells.

## Glutamine

Glutamine is a non-essential amino acid mainly transported into cells through SLC1A5 (also known as ASCT2) and SLC7A5 (also known as LAT1) transporters. Although glutamine is a non-essential amino acid, many cancers, including breast cancer, rely on the breakdown of glutamine to supplement the tricarboxylic acid cycle and synthesize glutathione (72–74). ASCT2 is identified as an individual prognostic marker for breast cancer patients (75, 76). It is found that missense mutant p53 oncoprotein stimulates essential amino acid intake by inducing the expression of serine-synthesis-pathway enzymes and L-type amino acid transporter 1 (LAT1)/CD98 heavy chain heterodimer, promoting breast cancer growth. This effect is exacerbated by amino acid deficiency, representing a mutant p53-dependent metabolic adaptive response. In the absence of amino acids, mutant p53 protein is stabilized and induces metabolic changes and amino acid transcription programs that maintain cancer cell proliferation (77).

SLC1A5 has been shown to be up-regulated during T cell activation, resulting in increased glutamine uptake (78). Deprivation of glutamine blocks the proliferation of T cells and the production of cytokines in an *in vitro* culture system (79). Reducing the consumption of glutamine by tumor cells or increasing the level of glutamine in the tumor microenvironment may improve the efficacy of tumor immunotherapy.

## Cystine

Cysteine is not an essential amino acid, but it is necessary for the synthesis of proteins, glutathione, and coenzyme A. In the extracellular matrix, cysteine usually exists in the form of oxidized cystine and is transported into cells through the glutamate-cystine antiporter system xCT transporter. Once transported into cells, cystine is immediately reduced to cysteine. The xCT transporter is expressed on one-third of TNBC samples. Ambient glutamine indirectly supports environmental cystine acquisition via the xCT cystine/glutamate antiporter (SLC7A11). Inhibition of xCT by clinically approved anti-inflammatory drug sulfasalazine can restrain tumor growth, revealing a therapeutic target in breast tumors of poorest prognosis (80). Moreover, targeting xCT can enhance the chemosensitivity of breast cancer stem cells to doxorubicin *in vivo*, indicating that xCT immunotargeting may be an effective adjuvant to chemotherapy (81, 82).

Exogenous cysteine or cystine is not necessary for early activation of human T cells cultured *in vitro*, but it is necessary for T cell expansion. *In vivo* observations have found that antigen-presenting cells, including dendritic cells, can transport cysteine to support T cell proliferation, while myeloid-derived suppressor cells can sequester extracellular cysteine and cystine to block T cell activation. Although SLC7A11-deficient mouse T cells cannot proliferate *in vitro*, they can be fully activated *in vivo*. In addition, *in vivo* experiments have confirmed that deletion of SLC7A11 gene has no effect on the anti-tumor response of T cells, but knockout of SLC7A11 gene in tumor cells can improve the efficacy of immunotherapy (83), which may be related to the decreased antioxidant activity of tumor cells and the increased cystine level in the microenvironment. A similar study in breast cancer has also suggested that xCT is dispensable for the normal functioning of the immune system, thus supporting the safety of xCT targeting in breast cancer (82).

## Arginine

Arginine is dispensable for the growth of breast cancer and it has two products, ornithine and nitric oxide (NO). Extracellular arginine is transported across the membrane through the y+ system of cationic amino acid transporters, including SLC7A1, SLC7A2, and SLC7A3. Arginine-succinate lyase (ASL) is the enzyme responsible for the production of arginine. Down-regulation of ASL inhibits the growth of breast cancer *in vitro* and *in vivo*, accompanied by a delay in G2/M transition (84). Arginine can produce NO through nitric oxide synthase (NOS). The high activity of inducible NOS (iNOS) is associated with the low survival rate of breast cancer patients (74). The production of NO can activate multiple carcinogenic signaling pathways. The NO signaling can also trigger the up-regulation of stem cell marker CD44 and other proteins with basal-like breast cancer (BLBC) characteristics, thereby promoting epithelial-mesenchymal transition (EMT), chemotherapy resistance, and invasion (85–87).

Arginine metabolism has an impact on T cells. Knockout of SLC7A1 by gene editing can reduce the arginine uptake of T cells, thereby inhibiting T cell proliferation. Additionally, arginine starvation can induce T cell cycle arrest, leading to the loss of the Zeta chain, a component of the T cell antigen receptor (TCR), and reducing T cell proliferation and cytokine production (88). In mouse models, exogenous arginine supplementation promotes the generation of central memory T cells, thereby enhancing CD8^+^ T cell-mediated anti-tumor activity. It has also been reported that arginine can enhance CD8^+^ T cell activation and anti-tumor responses by enhancing lymphocyte-specific protein tyrosine kinase (LCK) signaling (89). In addition to being used for protein synthesis, arginine is also metabolized to produce many substances, including nitric oxide, proline, ornithine, creatine, agmatine, and polyamines. The catabolism of arginine produces nitric oxide and its derivative peroxynitrite, which can inhibit T cell anti-tumor responses (90).

## Tryptophan

**Figure 4:**
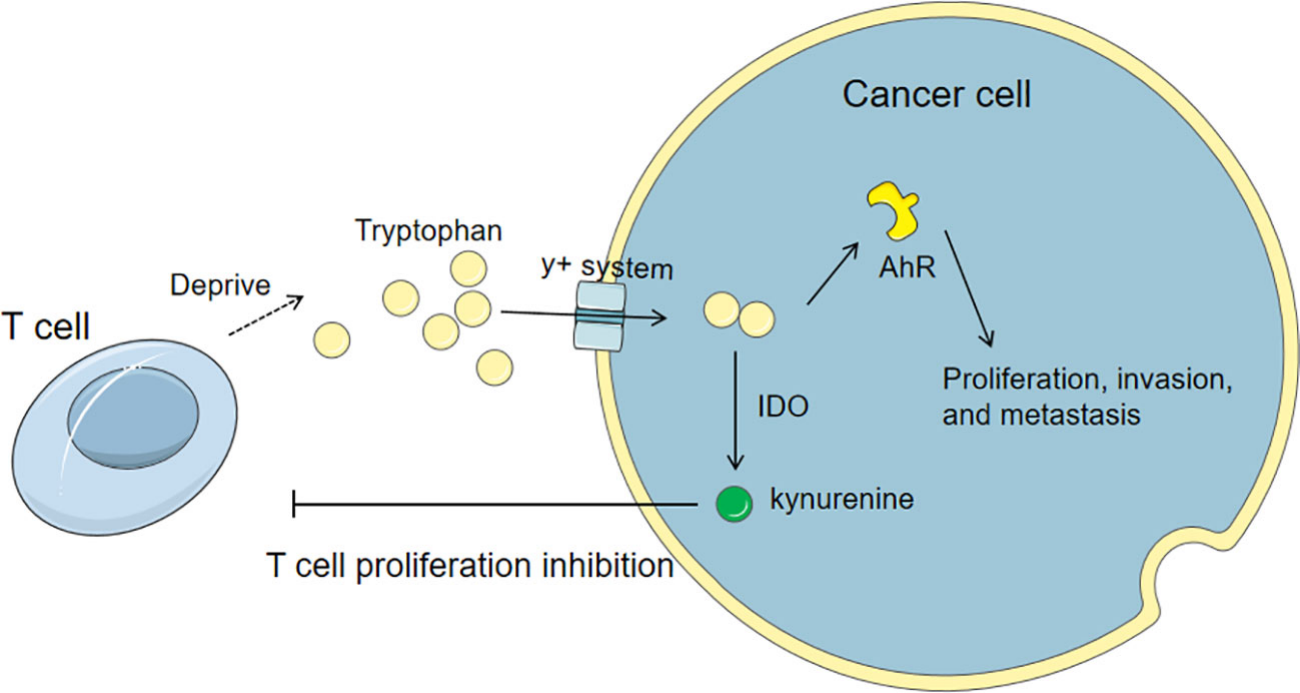
Tryptophan metabolism in cancer cells and its influence on T cells. Cancer cells up-regulate tryptophan transporters and competitively uptake tryptophan from the microenvironment. The tryptophan can activate AhR to promote the proliferation, invasion, and metastasis of cancer cells and directly inhibit the anti-tumor activity of T cells. Also, the catabolism of tryptophan via IDO produces kynurenine, which can directly inhibit the proliferation of T cells.

Tryptophan is an essential amino acid. Extracellular tryptophan is transported into cells through the neutral amino acid transport system L, and a small amount of intracellular tryptophan is used for protein synthesis and production of tryptamine and serotonin. More than 95% of free tryptophan is catalyzed by indoleamine-2,3-dioxygenase 1 (IDO1), IDO2, or tryptophan-2,3-dioxygenase (TDO) to produce kynurenine (Kyn). IDO1 and TDO are highly expressed in tumor cells, tumor stromal cells, dendritic cells, and macrophages in the tumor microenvironment, leading to the consumption of tryptophan and the accumulation of tryptophan-related metabolites (91, 92). These enzymes are aberrantly expressed in breast cancer. TDO2-produced tryptophan can increase the activation of aryl hydrocarbon receptor (AhR) in TNBC, thereby promoting the proliferation, invasion, and metastasis of cancer cells and directly inhibiting the anti-tumor activity of T cells (93–95). In addition, 5-hydroxytryptamine (5-HT), a metabolite of tryptophan, promotes the invasion and proliferation of TNBC cells through the 5-HT7 receptor, and increases the expression of tryptophan hydroxylase 1 (TPH1) and vascular endothelial growth factor (VEGF) (96) as shown in **Figure 4**.

## Lipid metabolism

As one of the three primary energy sources, lipids provide energy for all cellular life activities. Additionally, lipids are essential cellular components forming membranes, serving as energy reserves, and in some cases, acting as intra-extracellular signals. Cancer cells also harness lipid metabolism to modulate the activity of stromal and immune cells to resist therapy (97). Breast cancer remains the second leading cause of cancer-related deaths in female subjects mostly due to the considerable therapeutic resistance (98). Recent studies have established that cancer cells reprogram their lipid metabolism to develop into resistant phenotypes, indicating that targeting lipid reprogramming is a promising anticancer strategy (94–96).

The metabolic reprogramming of T cells in the immune microenvironment also involves changes in lipid metabolism patterns. CD8^+^ T cells including effector T cell subsets and memory T cell subsets have different lipid metabolism patterns (99, 100). Hypoxia in the tumor microenvironment induces effector T cells to undergo metabolic switching from oxidative phosphorylation to glycolysis, leading to more carbohydrate and lipid consumption. On the other hand, the differentiation and function of memory T cells mainly depend on mitochondrial fatty acid oxidation and oxidative phosphorylation to generate energy. Lipid metabolism is highly dependent on the transcription factors SREBP1 and SREBP2, which regulate the *de novo* synthesis of fatty acids, as well as the synthesis, uptake, and excretion of cholesterol (101). These transcription factors are essential for the effector response of CD8^+^ T cells because membrane synthesis requires lipids and cholesterol.

The regulation of cholesterol metabolism in CD8^+^ T cells is crucial for maintaining the required membrane fluidity during activation. The aggregation of TCR requires membrane fluidity, and TCR activation domains accumulate cholesterol, sphingomyelin, and saturated phosphatidylcholine, comprising a distinct molecular lipid composition (102). Inhibiting the cholesterol esterification enzyme acetyl-CoA acetyltransferase (ACAT1) can increase membrane cholesterol in CD8^+^ T cells, thereby improving TCR aggregation and signal transduction, and enhancing CD8^+^ T cell effector function and proliferation. Since the activation and expansion of T cells require cholesterol uptake and *de novo* synthesis, the efflux of cholesterol can limit T cell proliferation. T cell activation induces the expression of the sulfotransferase family 2b member 1 (Sult2b1), which promotes the sulfation of oxidized sterols, thereby reducing the activation of liver X receptors (LXR) and ultimately repressing the activation of the cholesterol efflux-related pathway ABCG1. The Sult2b1-LXR-ABCG1 axis promotes T cell proliferation by reducing the efflux of cellular cholesterol (103).

Proprotein convertase subtilisin/kexin type 9 (PCSK9) can impair the ability of the liver to process low-density lipoprotein cholesterol (LDL-C) in the blood by binding to low-density lipoprotein receptors (LDL-R) and interfering with their recycling, leading to cholesterol buildup in the blood and eventually cardiovascular diseases. Genetic deletion or pharmacological inhibition of PCSK9 in tumor cells can enhance the antitumor activity of CD8+ T cells. Mechanistically, inhibiting PCSK9 increases the expression of major histocompatibility protein class I (MHC I), promoting robust intratumoral infiltration of cytotoxic T cells (104, 105). The PCSK9-targeted drug MK-0616 is currently undergoing two phase 3 clinical trials to evaluate its effect on LDL-C reduction.

Fatty acid synthesis in T cells is highly dependent on acetyl-CoA carboxylase 1 (ACC1) to initiate the production of long-chain fatty acids. Compared with SREBP1-deficient T cells, ACC1-deficient T cells show impaired cell proliferation. During antigen-specific responses, CD8^+^ T cell-specific deletion of ACC1 results in lower survival rates in naive mice due to limited lipid molecules for clonal expansion (106). Carnitine palmitoyltransferase 1A (CPT1A) controls mitochondrial fatty acid oxidation by promoting the acylation of long-chain fatty acids and plays an important role in the production of CD8^+^ memory T cells (107). Developing novel therapeutic strategies by focusing on the unique role of fatty acid metabolism in tumor microenvironments of T cells has become a current consideration **Table 1**.

**Table 1:** Upregulated metabolic genes in breast cancer.

Metabolism	Genes	Function of genes	Ref
Glucose metabolism	GLUT1	Glucose uptake	(32, 33)
	GLUT4	Glucose uptake	(38)
	LDHA	catalyzes pyruvate to lactate	(52)
	LDHB	catalyzes pyruvate to lactate	(53)
	MCT1	Lactic acid transport	(63)
	MCT2	Lactic acid transport	(63)
	MCT4	Lactic acid transport	(63)
Amino Acid metabolism	SLC1A5	Glutamine uptake	(75, 76)
	SLC7A5	Glutamine uptake	(77)
	SLC7A11	cystine/glutamate antiporter	(80)
	ASL	Arginine catabolism	(84)
	IDO1	Arginine catabolism	(91, 92)
	TDO	Arginine catabolism	(91, 92)
Lipid metabolism	SREBP1	*de novo* synthesis of fatty acids	(101)
	SREBP2	*de novo* synthesis of fatty acids	(101)

## Metabolite mimetics

Metabolite mimetics possess profound and multifaceted importance as potential anticancer therapeutic pathways. Metabolite mimetics are compounds designed to mimic the biological effects of natural metabolites within the cellular environment, often targeting specific metabolic processes or enzymes critical for cancer cell survival and proliferation. Metabolite mimetics are increasingly considered as specific targets for cancer therapy. Cancer cells often display altered metabolic patterns to support their rapid growth and survival. By targeting these specific metabolic vulnerabilities, metabolite mimetics can potentially selectively kill cancer cells while minimizing damage to healthy tissues. The regulation of the metabolic landscape within this environment by metabolite mimetics can influence immune cell function and thereby promote anti-tumor immune responses (108). As discussed above, 2DG as an analog of glucose can affect tumor cells and anti-tumor immune functions. Metabolite mimetics may offer new therapeutic targets for cancer patients who develop resistance to current treatments or have limited therapeutic options. Briefly, the exploration of metabolite mimetics targeting specific pathways can broaden the range of anticancer treatment options and improve the outcomes for patients with difficult-to-treat cancers.

## Conclusion

Immunotherapy has revolutionized the management of multiple solid and hematologic malignancies. Nevertheless, the overall response rate of immunotherapy still left much to be desired, and some breast cancer patients initially receiving immunotherapy are prone to develop acquired resistance over time. Hence, it is urgent for clinical research to find new targets to improve the immunotherapeutic effect of breast cancer. The function of immune cells requires the coordination of metabolic patterns, and the metabolism of tumor cells and related cells has a significant impact on the immune environment. Breast cancer has its unique metabolic reprogramming, and combined with metabolic therapy, the immunosuppression of breast cancer can be improved.

Tumor and immune cells in the same environment need to maintain their metabolism to survive. The new targets should enhance immune effects without promoting tumor growth. The screening of effective biomarkers and the development of new drug targets require accurate detection of metabolites in the body, which involves the dynamic monitoring of the changes in tumors, microenvironment, and immune cells. Drug development requires a balance between efficacy and safety and compares the roles of different metabolic pathways in tumor immunity, thereby developing personalized strategies based on mutations and metabolic status.

## Author contributions

JZo: Investigation, Methodology, Writing – original draft. CM: Conceptualization, Data curation, Project administration, Validation, Writing – original draft. ZL: Project administration, Resources, Validation, Visualization, Writing – review & editing. JZh: Funding acquisition, Supervision, Writing – review & editing. GL: Project administration, Supervision, Writing – original draft, Writing – review & editing.
